# Functioning of Long Noncoding RNAs Expressed in Macrophage in the Development of Atherosclerosis

**DOI:** 10.3389/fphar.2020.567582

**Published:** 2020-11-26

**Authors:** Xirui Ma, Huifang Liu, Fengling Chen

**Affiliations:** Department of Endocrinology and Metabolism, Shanghai Ninth People’s Hospital, Shanghai Jiao Tong University School of Medicine, Shanghai, China

**Keywords:** long noncoding RNA, atherosclerosis, macrophage, kappa B, foam cell macrophage

## Abstract

Chronic inflammation is part of the pathological process during atherosclerosis (AS). Due to the abundance of monocytes/macrophages within the arterial plaque, monocytes/macrophages have become a critical cellular target in AS studies. In recent decades, a number of long noncoding RNAs (lncRNAs) have been found to exert regulatory roles on the macrophage metabolism and macrophage plasticity, consequently promoting or suppressing atherosclerotic inflammation. In this review, we provide a comprehensive overview of lncRNAs in macrophage biology, highlighting the potential role of lncRNAs in AS based on recent findings, with the aim to identify disease biomarkers and future therapeutic interventions for AS.

## Introduction

Atherosclerosis (AS) is a multifaceted chronic inflammatory disease characterized by the formation of atherosclerotic plaques predominantly at branch points of arteries and bifurcations due to the disturbed laminar flow at these sites (Shapouri-Moghaddam et al., 2018). Atherosclerotic plaques consist of lipids, foam cells, calcified sites, and necrotic cores (Donaldson et al., 2018; Moore et al., 2013; Shapouri-Moghaddam et al., 2018; Tabas and Bornfeldt, 2016). Monocytes and macrophages are dynamically involved in the initiation and development of AS and ultimately contribute to plaque rupture. An altered metabolism dictates macrophage activities and subsequent AS progression (Koelwyn et al., 2018).

During early atherogenic stages, apolipoprotein B-lipoproteins accumulated in the intima initiate an early inflammatory response and formation of fatty streak lesions (Williams and Tabas, 1995; Moore and Tabas, 2011; Liu et al., 2014). The inflammatory response progresses through various modifications in the endothelium, such as oxidation (causing altered expression of adhesion molecules and elevating secretion of chemokines) (Moore and Tabas, 2011). Activated endothelial cells produce monocyte chemoattractant protein-1 (MCP-1). On monocytes, MCP-1 can interact with cognate chemokine receptors and promote monocyte migration in a specific direction (Moore and Tabas, 2011). Later, the recruited monocytes are tethered and roll along the endothelium. The interaction between P-selectin glycoprotein ligand-1 molecules on monocytes and endothelial selectins further triggers firm adhesions (Mestas and Ley, 2008). After entering the intima, monocytes gradually differentiate into macrophages and internalize native and modified lipoproteins (Johnson and Newby, 2009; Paulson et al., 2010). Atherosclerosis is a nonresolving inflammatory condition characterized by monocytes continually entering the intima and lesion plaques and constantly differentiating into macrophages (Moore and Tabas, 2011).

Lipid uptake, cholesterol esterification, and efflux are three distinct processes of a normal cholesterol metabolism in macrophages (Chistiakov et al., 2017). Disturbances of the cholesterol metabolism are a key contributor to AS, resulting in the accumulation of lipids in macrophages and the formation of “foam cells” (Crowther, 2005; Maguire et al., 2019). In the early plaque, uptake of modified low-density lipoprotein (LDL) (Kunjathoor et al., 2002), phagocytosis of matrix-retained LPs, and pinocytosis of fluid native LDL contribute to foam cell formation (Tabas et al., 1993; Kruth et al., 2005; Moore and Tabas, 2011). Key scavenger receptors such as the CD36, SR-A class, and lectin-like ox-LDL receptor-1 play regulatory roles on cholesterol uptake and the formation of foam cells (Moore and Freeman, uhu2006).

In advanced AS lesions, macrophage apoptosis, incomplete clearance, and defective phagocytosis of apoptotic macrophages give rise to necrotic cores, which exacerbate inflammation, incite thrombosis, and increase inner stress on the fibrous plaques (Virmani et al., 2002; Tabas, 2010). Notably, thinning of the fibrous cap and necrotic core size increments are critical features of vulnerable plaques. The sites on the shoulder of necrotic cores are vulnerable to rupture. Functions and biological mechanisms of all lncRNAs we discussed below are summarized in Table 1.

**TABLE 1:** Summary of atherosclerosis-related lncRNAs expressed in macrophage.

lncRNA	Full name	Location	Stimulus	Cells	Function	Mechanism	Reference
Macrophage differentiation							
Lnc-MC	Long noncoding monocytic RNA	Cytoplasm	Pam3CSK4	Macrophage HL-60	Monocyte/macrophage differentiation	Sequestering and soaking up miR-199a-5p to release the expression of ACVR1B	Chen et al. (2015)
Macrophage phenotypic switching							
TCONS_00019715	—	—	IFN-α LPS	Macrophage	Macrophage polarization	Reducing M1 elevating M2 may be through regulating PAK1	Huang Z. et al. (2016)
Mirt2	Myocardial infraction-associated transcript 2	Cytoplasm	LPS	Macrophage	Macrophage polarization and anti-inflammation	Blocking the MyD88-mediated MAPK and NF-κB activities to inhibit M1 polarization	Du et al. (2017)
Macrophage apoptosis							
CERNA1	—	Cytoplasm and nucleus	Ox-LDL	Vascular smooth muscle cells and macrophage	Atherosclerotic plaque stabilization	Increasing API5 to inhibit apoptosis of VSMCs and anti-inflammatory macrophages	Lu et al. (2019)
RAPIA	LncRNA associated with the progression and intervention of atherosclerosis	Cytoplasm	—	Macrophage	Macrophage proliferation and apoptosis	Binding to miRNA-183-5p to promote proliferation and promote apoptosis of macrophages	Sun et al. (2020)
Macrophage pyroptosis							
MALAT1	Metastasis-associated lung adenocarcinoma transcript 1	Nucleus	Low-dose sinapic acid	Macrophage	Macrophage pyroptosis	Chronic low-dose SA treatment could block the function of MALAT1-dependent NLRP3 inflammasome, consequently inhibit pyroptosis, and systemic inflammatory response	Yong et al. (2018)
Atherosclerotic inflammation							
Mirt2	Myocardial infraction-associated transcript 2	Cytoplasm	LPS	Macrophage	Macrophage polarization and anti-inflammation	Blocking the MyD88-mediated MAPK and NF-κB activities to inhibit M1 polarization	Du et al. (2017)
LINC00305	—	Cytoplasm	LPS	Macrophage	Pro-inflammation	Binding to LIMR then promoted nuclear localization of AHRR to activate NF-κB	Zhang et al. (2017)
LIN28B-AS1	—	Nucleus	LPS	Macrophage and monocyte	Pro-inflammation	Increasing IGF2BP1-p65-p52 association to activate NF-κB signaling	Xie Z. et al. (2019)
MALAT1	Metastasis-associated lung adenocarcinoma transcript 1	Nucleus	LPS	Macrophage	Decreased transcription of inflammatory cytokines	Interacting with NF-κB p50/p65 dimmers to sequester NF-κB and decrease cytokine transcription	Zhao et al. (2016)
SNHG16	Small nucleolar RNA host gene 16	Cytoplasm	Ox-LDL	Macrophage	Cell proliferation and pro-inflammation	Binding to and absorbed miR-17-5p to release the activity of NF-κB pathway	An et al. (2019)
LINC01140	—	—	Ox-LDL	Macrophage	Anti-inflammation	Binding to miR-23b in order to down-regulate p53 and decrease the expression of inflammation factors	He et al. (2020)
Dnm3os	Dynamin 3 opposite strand	Nucleus	PA and HG	Macrophage	Pro-inflammation	Decreasing nucleolin protein levels to increase inflammatory gene expression	Das et al. (2018)
lincRNA-EPS	LincRNA erythroid prosurvival	Nucleus	Pam3CSK4	Macrophage	Anti-inflammation	Controlling nucleosome positioning and repressing transcription expression of inflammatory cytokines by targeting hnRNPL	Atianand et al. (2016)
uc.48+	—	—	HG and FFAs	Macrophage	Pro-inflammation and promote immune response	Evoking P2X7R-mediated cytokine production, ROS activity, and reaction of the ERK pathway	Wu et al. (2018)
Macrophage cholesterol metabolism							
NEAT1	Nuclear-enriched abundant transcript 1	Nucleus	Ox-LDL	Macrophage	Inflammation and oxidative stress	Sponging and inhibiting miR-128 to trigger inflammation and oxidative stress and to increase CD36	Chen et al. (2018)
			Ox-LDL	Macrophage	Inflammation and lipid uptake	Inducing ox-LDL-induced apoptosis and inflammation via targeting miR-342-3p	Wang L. et al. (2019)
GAS5	Growth arrest–specific transcript 5	Cytoplasm	Ox-LDL	Macrophage	Atherosclerotic plaque destabilization	Suppressing the miR-211 expression to aggravate inflammatory response and the expression of MMP exacerbating plaque rupture	Ye et al. (2018)
		Nucleus	Ox-LDL	Macrophage	Pro-atherosclerotic	Promoting atherosclerosis development through targeting EZH2-mediated ABCA1 transcription	Meng et al. (2020)
RP5-833A20.1	—	Nucleus	Ox-LDL and Ac-LDL	Macrophage-derived foam cells	Anti-atherosclerotic	Reducing cholesterol efflux and alleviating inflammatory responses via RP5-833A20.1-has-miR-382-5p pathway	Hu et al. (2015)
ZFAS1	Zinc finger NFX1-type containing 1 antisense RNA 1	Cytoplasm	Ox-LDL	Macrophage-derived foam cells	Anti-atherosclerotic	Ameliorating inflammation and reducing cholesterol efflux by targeting miR-654-3p-ADAM10/RAB22A axis	Tang et al. (2020)
CHROME	Cholesterol homeostasis regulator of miRNA expression	Cytoplasm	LXR agonist (GW3965	Hepatocytes and macrophage	Anti-atherosclerotic	Interacting with microRNAs to curb their repression on cholesterol efflux and HDL biogenesis	Hennessy et al. (2019)
H19	H19-imprinted maternally expressed transcript	Nucleus	Ox-LDL	Macrophage	Anti-atherosclerotic	Regulating adipogenesis and inflammation by inhibiting miR-130b	Han et al. (2018)
PELATON/LINC01272	Plaque-enriched lncRNA in atherosclerotic and inflammatory bowel macrophage regulation	Nucleus	—	Macrophage	Regulator of phagocytosis	Inducing CD36 expression to promote phagocytosis, ROS production, and ox-LDL uptake	Hung et al. (2019)
TUG1	Taurine up-regulated gene 1	—	Ox-LDL	Macrophage	Pro-atherosclerotic	Dysregulating high-density lipoprotein metabolism and cholesterol efflux through inhibiting miR-92a and improving FXR1	Yang and Li (2020)
E330013P06	—	—	HG PA	Macrophage	Pro-atherogenic	Increasing inflammatory genes along with foam cell formation through up-regulating CD36 expression	Reddy et al. (2014)
lincRNA-DYNLRB2-2/LINC01228	—	—	Ox-LDL	Macrophage	Anti-atherosclerotic	Up-regulating GPR 119 and ABCA1 by the GLP1-R signaling pathway	Hu et al. (2014)
MeXis	Macrophage-expressed LXR-induced sequence	Nucleus	GW3965	Macrophage	Anti-atherosclerotic	Promoting LXR-mediated Abca1 expression and cholesterol efflux	Sallam et al. (2018)
HOXC-AS1	HOXC cluster and antisense RNA 1	—	Ox-LDL	Macrophage	Regulation of cholesterol accumulation	Suppressing lipid accumulation by increasing HOXC6 expression	Huang C. et al. (2016)
CDKN2B-AS1/ANRIL	Cyclin-dependent kinase inhibitor 2B antisense noncoding RNA	Nucleus	Ox-LDL	Macrophage	Pro-atherosclerotic	Assembling an RNA–DNA triplex in the promoter region of CDKN2B to recruit EZH2 and CTCF, then increase histone methylation, then adversely affect EZH2-mediated gene transcription	Ou et al. (2020)
					Anti-atherosclerotic	Binding to DNMT1 to improve methylation of ADAM10 promoter to suppress inflammation and cytokine production, and promote cholesterol efflux	Li et al. (2019)

## Long Noncoding RNAs

Long noncoding RNAs (lncRNAs) are defined as a large class of non-protein-coding transcripts. They consist of more than 200 nucleotides in length. Knowledge on the biological function of lncRNAs has been expanding with new publications in the fields of epigenetic activity regulation (McHugh et al., 2015), *cis*- (Engreitz et al., 2016) and *trans*- (Atianand et al., 2016) gene transcription regulations, protein translation (Carrieri et al., 2012), RNA (Hansen et al., 2013) or protein “sponging,” (Tichon et al., 2016), and nuclear/cytoplasmic “shuttling” (Yap et al., 2018; Maguire and Xiao, 2020).

In accordance with the positional association between lncRNAs and protein-coding genes (Gao et al., 2020), the lncRNAs can be classified as exonic sense, anti-sense, intronic sense, bidirectional (enhancer), and intergenic sense classes (Derrien et al., 2012). At the transcriptional level, lncRNAs can also be divided into four models (signals, decoys, guides, or scaffolds) to regulate gene expression (Chang, 2011; Mathy and Chen, 2017; Wang and Chang, 2011).

## Long Noncoding RNAs in Macrophage Differentiation

Monocyte chemotaxis is triggered by chemokines or cytokines released from cells in damaged tissue or infection areas and stimulates monocytes to migrate to pathologic sites and begin differentiation into macrophages. These macrophages effectively take infections under control and remove dead cells and debris for tissue repair and wound healing. On the other hand, macrophages are involved in the inflammatory tissue damage caused by inflammatory diseases (Moore and Tabas, 2011).

In the development of AS, MCP-1 secreted by endothelial cells is a potent chemokine for monocyte migration involved in the initiation of the inflammatory response (Panee, 2012). MCP-1 attracts monocytes in the circulation, triggering migration via its interaction with the membrane CC chemokine receptor 2 on monocytes. Under normal blood flow conditions, monocytes can firmly adhere to the vascular endothelium through interactions with MCP-1, IL-8, or CXC ligand-8 (Melgarejo et al., 2009, Shapouri-Moghaddam et al., 2018; Moghaddam et al., 2018). After entering the intima, phagocytic monocyte-derived macrophages start to internalize native and modified lipoproteins (Johnson and Newby, 2009; Paulson et al., 2010; Moore and Tabas, 2011).

Long noncoding monocytic RNA (lnc-MC) and miR-199a-5p, both PU.1-regulated noncoding RNAs, work together during human monocyte/macrophage differentiation. The dominant transcription factor PU.1 commits the monocytic lineage during hematopoiesis and promotes the maturation of monocytes/macrophages (Anderson et al., 1998; Lin et al., 2014). PU.1 transcriptionally regulates lnc-MC. Increased expression of lnc-MC reinforces the role of PU.1 by sequestering and soaking upmiR-199a-5p, relieving the suppression on the expression of activin A receptor type 1B, an important regulator of monocyte/macrophage differentiation. This suggests that lnc-MC acts as an antagonist of miR-199a-5p and strengthens the role of PU.1 in cell differentiation (Chen et al., 2015).

## Long Noncoding RNAs in Macrophage Phenotypic Switching

Plasticity and polarization are hallmarks of macrophages (Wang L. X. et al., 2019). Phenotype switching of macrophages, in response to cues from the local microenvironment, is necessary for a diversity of indispensable functions during host defense responses and tissue repair (Koelwyn et al., 2018). Reciprocal skewing of macrophage polarization is modulated by many intricate factors like the daily dietary intakes and transcriptional factors (Chinetti-Gbaguidi and Staels, 2011). Macrophages can be broadly categorized into two types: M1 and M2 phenotypes. Classically, M1-activated macrophages enhance the production of pro-inflammatory cytokines (TNF, IL-6, IL-1β, IL-12, and IL-23 in humans) and lower the secretion of IL-10 (Verreck et al., 2004), whereas M2 macrophages are characterized by their improved endocytic clearance capacity that can protect local tissues from detrimental inflammatory damages and eliminate inflammation (Mantovani et al., 2004; Chinetti-Gbaguidi and Staels, 2011). Therefore, metabolic reprogramming in macrophages has a direct influence on cell functions and energy homeostasis (Koelwyn et al., 2018). In AS, both macrophage phenotypes are present in fibrous caps of established lesions (Anderson et al., 2002), with a predominance of M1 over M2 in progressing atherosclerotic lesions (Khallou-Laschet et al., 2010; Chinetti-Gbaguidi and Staels, 2011; Khallou et al., 2010). TCONS_00019715 was the first reported lncRNA expressed in human macrophages with phenotype switching functions. The expression level of TCONS_00019715 in macrophages was drastically induced by IFN-γ + LPS stimulation, and it underwent a strong reduction after IL-4 treatment. Knockdown of TCONS_00019715 reduced the expression of M1 markers in IFN-γ + LPS-stimulated macrophages, and it elevated the M2 phenotype markers in IL-4-stimulated ones. PAK1 (p21-activated kinase 1), an important protein-coding gene associated with TCONS_00019715, has been speculated to mediate TCONS_00019715’s macrophage polarization effects (Huang Z. et al., 2016). The myocardial infarction–associated transcript 2 (Mirt2) is another lncRNA that has been proved to affect macrophage phenotypic switching. It can block the expression of the M1 polarization and has anti-inflammatory functions through its control of NF-κB activation (Du et al., 2017). Detailed information about the function of Mirt2 will be covered in a later section.

## Long Noncoding RNAs in Macrophage Apoptosis

Macrophage apoptosis occurs during the atherosclerotic plaque development process. Apoptosis in the early stages is protective because it cleans up macrophage foam cells residing inside the plaque lesions (Shapouri-Moghaddam et al., 2018).

LincRNA-p21 has been shown to modulate cell proliferation and apoptosis in AS. In apolipoprotein E–deficient (ApoE^−/−^) mice with atherosclerotic plaques, lincRNA-p21 expression was dramatically reduced at the transcription level. Small interfering RNA–induced lincRNA-p21 inhibition greatly increased the total number of RAW274.7 and HA-VSMC cells. P53 could transcriptionally target lincRNA-p21. In the mouse carotid artery injury model, blockade of lncRNA-p21 dysregulated many p53 downstream targets, leading to neointimal hyperplasia and enhanced cell proliferation with reduced apoptosis (Wu et al., 2014).

Cytoplasmic lncRNA CERNA1 can stabilize atherosclerotic plaques by promoting an important apoptosis inhibitor named apoptosis inhibitor 5 (API5). API5 is capable of inhibiting apoptosis of VSMCs and anti-inflammatory macrophages in apolipoprotein E^−/−^ (Apo E^−/−^) mice (Lu et al., 2019).

In a most recent study, an lncRNA associated with the progression and intervention of AS named RAPIA was increasingly expressed in advanced atherosclerotic sites and in macrophages. Blockade of RAPIA greatly attenuated the development of advanced AS in ApoE^−/−^ mice (Sun et al., 2020). RAPIA exerted a regulatory role by targeting miR-183-5p in macrophages. miR-183-5p contains two binding sites for RAPIA. As RAPIA binds to miRNA-183-5p, miRNA loses its ability to inhibit proliferation or to promote macrophage apoptosis. Interestingly, suppression of RAPIA has atheroprotective effects in ApoE^−/−^ mice fed with a high-fat diet which is similar to those of atorvastatin on advanced atherosclerotic plaques, by attenuating lipid accumulation, decreasing plaque size, increasing collagen content, and decreasing macrophage accumulation in advanced atherosclerotic plaques. Therefore, repressing RAPIA expression may be an alternative treatment for advanced atherosclerotic lesions, especially in patients resistant or intolerant to statins (Sun et al., 2020).

## Long Noncoding RNAs in Macrophage Pyroptosis

Pyroptosis is a programmed cell death of macrophages controlled by the NF-ĸB pathway (Bergsbaken et al., 2009; Sunami et al., 2012; Shen et al., 2014; Sheng et al., 2014). It causes cell lysis. Inflammatory responses can be induced upon cytosolic content release to the extracellular space (Xu et al., 2018). However, this type of cell death, once mislabeled as apoptosis, is attributable to the involvement of caspase 1 (Shi et al., 2017). Pyroptosis can be induced in macrophages in lesions by ox-LDL and cholesterol crystal–triggered increases in NLRP3 inflammasome and caspase 1, leading to AS progression. In advanced atherosclerotic lesions, the formation of necrotic cores and unstable plaques may be attributed to macrophage pyroptosis (Xu et al., 2018).

MALAT1 in diabetic AS has been reported to participate in macrophage pyroptosis after sinapic acid (SA) treatment. Gain- and loss-of-function approaches have demonstrated that in normal macrophages, MALAT1 shows a modestly beneficial effect against pyroptosis. Chronic low-dose SA treatment could block the inflammasome activation, macrophage pyroptosis, and the systemic inflammatory response by mediating MALAT1 (Yong et al., 2018).

## Long Noncoding RNAs in Macrophage During Atherosclerotic Inflammation

Many studies have shown that lncRNAs affect the expression profiles of inflammatory pathways in different diseases. For example, in response to LPS stimulation, LIN28B-AS1 (Xie Z. et al., 2019) and Mirt2 (Du et al., 2017) associate with pro- and anti-atherosclerotic inflammation factors in macrophages through NF-κB. Mathy’s review provided detailed information about lncRNAs’ classification and functions in transcriptional control (Mathy and Chen, 2017). In this review, we focused on the functions and effects of individual lncRNAs in the context of atherosclerotic inflammation.

The canonical NF-κB signaling is critical for regulation of innate and adaptive immune responses and is involved in cell proliferation and apoptosis, migration, and invasion (Taniguchi and Karin, 2018). The activation of NF-κB is induced by inflammatory chemokines, notably LPS, TNF-α, IL-1, and many toll-like receptors (TLRs) (Qin et al., 2005; Mathy and Chen, 2017). Under inactive conditions, IκBα sequesters cytoplasmic p50-p65 dimers, hindering the translocation of p50-p65 dimers to the nucleus. After receiving activating signals, IκBα is phosphorylated by a IκB kinase complex and get degraded into free NF-κB dimers. Free NF-κB dimers translocate to the nucleus, where they interact with specific DNA-binding sites to augment transcription and expression of different genes (Baker and Ghosh, 2010; Hayden and Ghosh, 2014; Mitchell and Carmody, 2018).

A number of studies have mentioned lncRNAs’ diverse regulatory roles in inflammatory diseases, including AS, by controlling NF-κB–regulated transcription in both the cytosol and nucleus.

## Toll-Like Receptor Ligation-Stimulated Long Noncoding RNAs

In response to LPS stimulation, lncRNA-Mirt2 (Du et al., 2017), LINC00305, LIN28B-AS1 (Zhang et al., 2017), and MALAT1 (Zhao et al., 2016) show remarkable effects on the progression of AS through NF-κB–dependent mechanisms. LINC00305 is a pro-inflammatory agent during AS progression. In Zhang’s work, LINC00305 in the cytosol interacted with the transmembrane protein lipocalin-1 interacting membrane receptor (LIMR) and promoted the expression of inflammatory genes in human THP-1 cells. Notably, LINC00305 has been associated with LPS-stimulated inflammation by targeting the transmembrane receptor LIMR. The inflammation promotor in LPS shock, aryl-hydrocarbon receptor repressor (AHRR), is a binding partner of LIMR (Brandstätter et al., 2016) that tends to enhance NF-κB activity when cotransfected with LIMR. LINC00305-LIMR interaction strengthened LIMR–AHRR binding and promoted AHRR nuclear localization in addition to promoting NF-κB activation, which in turn inhibited the downstream aryl-hydrocarbon receptor signaling (Zhang et al., 2017).

In the nucleus, a novel insulin-like growth factor 2 mRNA-binding protein 1 (IGF2BP1)–binding lncRNA (LIN28B-AS1) has been associated with pro-inflammatory activity. TLR4 ligation activates canonical NF-κB pathways, followed by release and activation of p65-p52 heterodimers. The p65–p52 heterodimers then shuttle to the nucleus and enhance pro-inflammatory responses, including facilitating gene transcriptions of IL-6, IL-1β, and TNF-α by interacting with IGF2BP1 (Perkins, 2007; Rahman and McFadden, 2011; Xie J. et al., 2019). It is noteworthy that LIN28B–AS1–IGF2BP1 binding is essential for IGF2BP1–p65–p52 complex formation, because nuclear LIN28B-AS1 could interact with IGF2BP1 and assemble the IGF2BP1-p65-p52 complex in THP-1 cells (Xie Z. et al., 2019).

Unlike LIN28B-AS1, anti-inflammatory lncRNA-Mirt2 regulates inflammation by blocking the NF-κB signaling in the cytosol. Mirt2 expression was potently up-regulated in response to LPS. LPS-triggered signaling pathways required the adaptor protein myeloid differentiation marker 88 (MyD88) and toll-interleukin-1 receptor domain–containing adaptor-inducing IFNβ (TRIF) (Kawai and Akira, 2010). The TLR4-MyD88 binding at the membrane contributed to recruiting and phosphorylating IL-1 receptor associated kinase 1 (IRAK1) and IRAK4 that promote oligomerization and ubiquitination of TNF receptor-associated factor 6 (TRAF6) (Hirotani et al., 2005; Skaug et al., 2009). However, Mirt2 can bind to TRAF6 in the cytosol and attenuate oligomerization and its Lys63 (K63)-linked ubiquitination, which restricts MyD88-dependent NF-κB and MAPK activation and TRAF6-mediated M1 polarization of macrophages. In addition to repressing macrophage inflammation, exotic Mirt2 expression remarkably facilitates IL-4–stimulated expression of multiple M2 markers, including Arg1, CD206, and Ym1, suggesting Mirt2 participates in M2 polarization. But the underlying mechanism remains unclear, and more studies are needed to gain a deeper understanding of the mechanism by which Mirt2 facilitates M2 polarization (Du et al., 2017).

Several findings have confirmed MALAT1 as a key controller of inflammation. Following LPS treatment, NF-κB–dependent enhancement of MALAT1 expression initiates a negative feedback loop. In human THP-1 cells, nuclear NF-κB triggered the transcription of MALAT1, and after that, MALAT1 interacted with the p50/p65 complex sequestering NF-κB and repressing its DNA-binding activity, which subsequently led to transcription of inflammatory cytokines TNF-α, IL-6, and IL-1β. MALAT1 knockdown enhanced the binding ability of p65 to TNF-α and IL-6 promoters (Zhao et al., 2016).

## Ox-LDL-Stimulated Long Noncoding RNAs

In addition to responding to TLR4 ligation, MALAT1 facilitates ox-LDL–induced inflammation by controlling CD36 expression. CD36 on the membrane of macrophages is a key scavenger receptor participating in lipid uptake and forming foam cells, and it has a strong affinity for ox-LDL (Tarhda et al., 2013; Chistiakov et al., 2017). Ox-LDL stimulation initiates MALAT1 transcription via NF-κB. *ß*-catenin is a transcription factor for CD36 expression, and *ß*-catenin gets recruited to the binding site on the CD36 promotor by the enhanced expression of MALAT1 that facilitates lipid uptake in macrophages (Huangfu et al., 2018).

SNHG16, an ox-LDL-sensitive long noncoding small nucleolar RNA, was found to be highly expressed in patients with AS. In an *in vitro* study, exogenous SNHG16 expression augmented production of pro-inflammatory cytokines by activating NF-κB signaling cascades. Conversely, SNHG16 knockdown resulted in inhibited IKKβ expression, IκBα phosphorylation, and p65 phosphorylation. Functionally, SNHG16 gets released into the cytoplasm in response to ox-LDL; then, SNHG16 acting as a sponge binds directly to and absorbs miR-17-5p to abolish the inhibitory effect of miR-17=5p on NF-κB activation. A SNHG16/miR-17-5p/NF-κB signaling axis promoting an inflammatory response in AS may exist (An al., 2019).

LINC01140 mediates ox-LDL–induced inflammation and plays a protective role on inflammation (He et al., 2020). After ox-LDL stimulation, LINC01140 becomes down-regulated in macrophage-differentiated THP1 cells, whereas p53 mRNA and miR-23b are up-regulated. The expression of inflammation factors, such as MCP-1, TNF-α, and IL-1β, is repressed potently when p53 is down-regulated. The association between p53 and the LINC01140 promoters was shown using a luciferase reporter assay. After transfecting cells with a p53-overexpression vector (pcDNA-p53), LINC01140 expression was repressed and miR-23b expression was enhanced (He et al., 2020).

## High Glucose and Palmitic Acid-Stimulated Long Noncoding RNAs

High glucose (HG) and palmitic acid (PA) can activate the lncRNA dynamin 3 opposite strand (Dnm3os) promotor and enhance pro-inflammatory actions by targeting nucleolin. Gene overexpression and knockdown experiments identified Dnm3os as a pro-inflammatory molecule under diabetic conditions. For instance, PA significantly induces the expression of IL-6, TNF, Nos2, and Cd36 in RAW264.7 cells transfected with a Dnm3os expression vector. RNA pull-down assays identified a close interaction between Dnm3os and nucleolin. Nucleolin is a nuclear RNA-binding protein in macrophages (Cong et al., 2011) that can regulate chromatin structure and exert an atheroprotective function. HG and PA substantially decrease the nucleolin protein level. Silencing nucleolin increased the gene expression of inflammatory factors induced by Dnm3os, including that of IL-6 and histone H3K9-acetylation at their promoters (Das et al., 2018).

In addition to activating lncRNAs through NF-ĸB, lincRNA-EPS, for example, is a nuclear repressor for inflammation that controls the expression of immune response genes (IRGs). Under an endotoxin challenge, lincRNA-EPS–deficient mice displayed exacerbated inflammatory responses and lethality. Gain-of-function and rescue approaches showed that lincRNA-EPS overexpression effectively limited IRG expression. lincRNA-EPS localizing at regulatory sites of IRGs interacts with hnRNPL via a CANACA motif to control nucleosome positioning and repress IRG transcriptions (Atianand et al., 2016).

On the other hand, lncRNA uc.48+ modulates P2X7R-mediated diabetic immune and inflammatory responses in RAW264.7 macrophages. P2X7R is protein of the P2X7 receptor and ERK pathway (Ponnusamy et al., 2011; Zanin et al., 2015). HG and free fatty acid (FFA) RAW264.7 cell treatments promoted the expression of uc.48+. Increased expression of uc.48+ in response to HG and FFAs augmented the inflammatory cytokine secretion, ROS formation, and ERK signaling via P2X7R activation (Wu et al., 2018).

**FIGURE 1:**
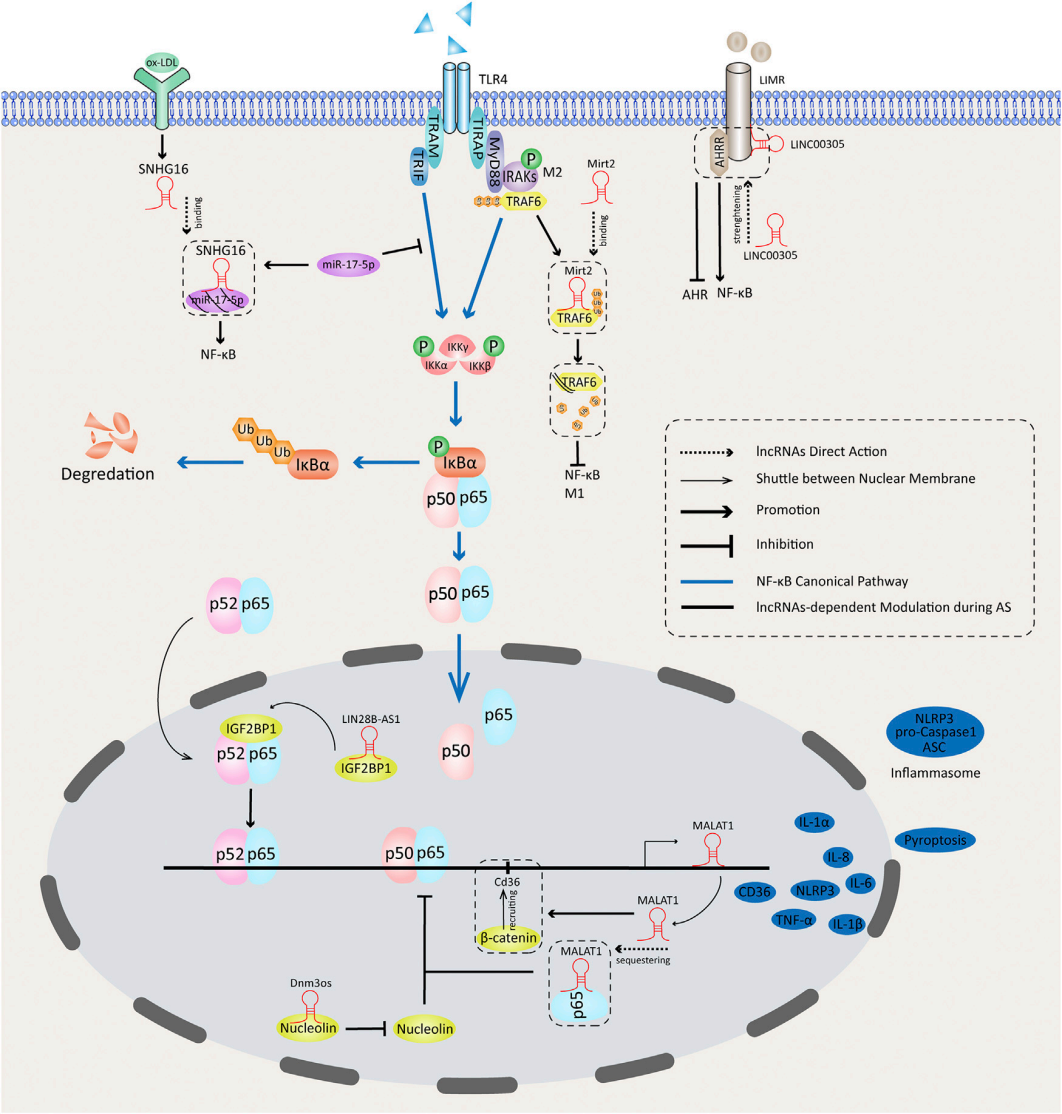
Functions of NF-κB–dependent lncRNAs in atherosclerotic inflammation.

Figure 1 summarizes the NF-κB–associated activities of lncRNAs during AS in macrophages. lncRNAs regulate the expression of NF-κB in both cytosols and nuclei. They can promote or inhibit translocation of NF-κB dimers to the nucleus by controlling the phosphorylation of the IKK complex and IκBα. NF-κB–dependent gene transcriptions are functionally affected by lncRNAs in the nucleus, partially through assembly of functional nuclear proteins complexes. MALAT1 can be expressed through an NF-κB pathway, and it affects the transcriptions of NF-κB–dependent inflammatory genes.

## Long Noncoding RNAs in Macrophage Cholesterol Metabolism

**FIGURE 2:**
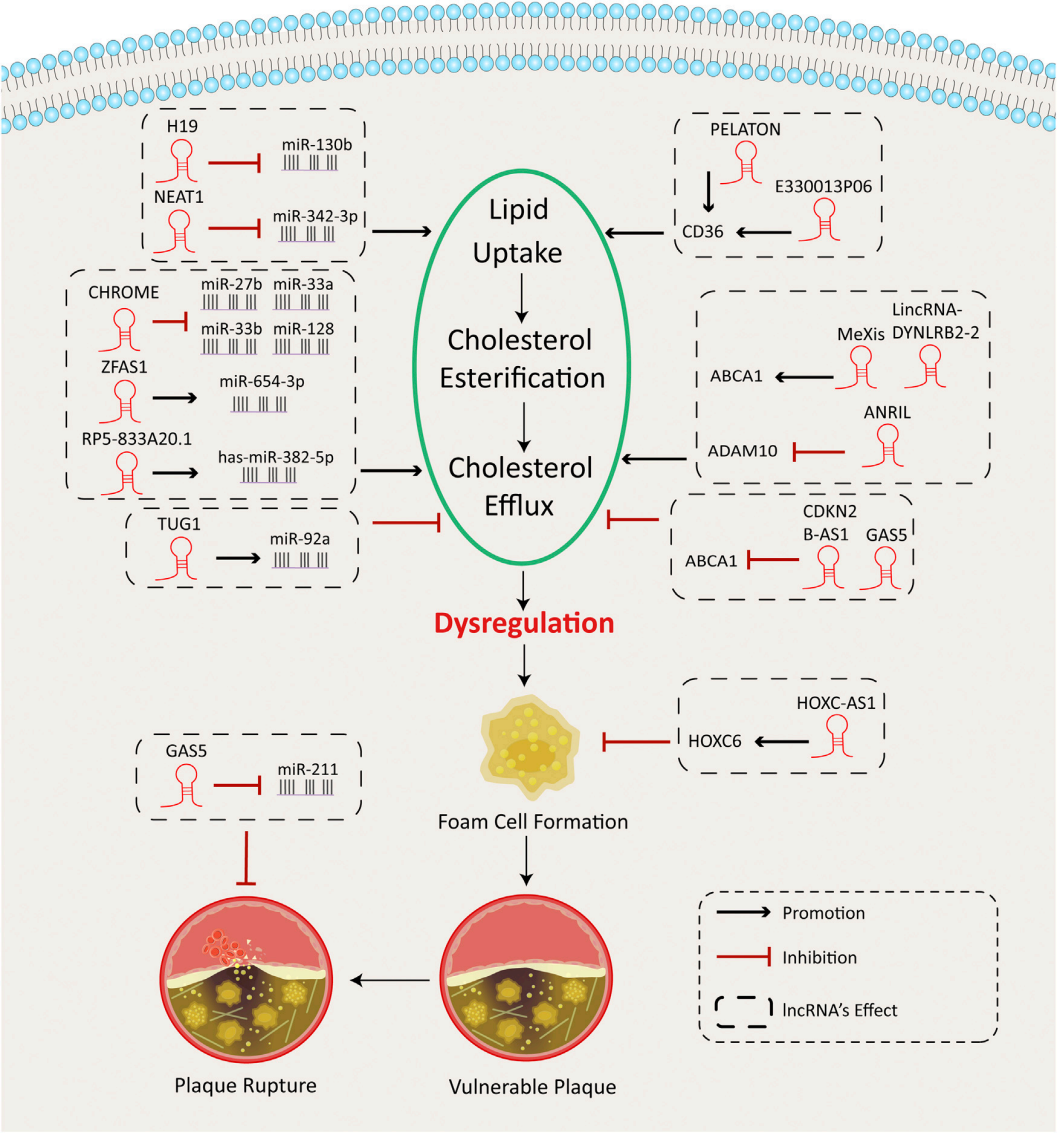
Cholesterol metabolism functions of lncRNAs in macrophages.

Lipid uptake and foam cell formation depend on activation of scavenger receptors, including the type A scavenger receptor (SRA) and the type B CD36 in the macrophages (Kunjathoor et al., 2002). Following lipid uptake, lipid droplets bud off the endoplasmic reticulum (ER) into the cytoplasm. The accumulation of free cholesterol (FC) requires re-esterification by the enzyme acyl-cholesterol transferase 1 (ACAT-1) in cells. Excessive FC can be stored in the form of cholesterol esters (CE) (McLaren et al., 2011; Maguire et al., 2019). Furthermore, the efflux of cholesterol can potentially keep cells from foaming. Cholesterol efflux depends mainly on several membrane transporters, such as the ATP-binding cassette transporter 1 (ABCA1) and the ATP-binding cassette subfamily G member-1 (ABCG1); and SR-B1, PPAR, and liver X receptor α (LXLRα) are key transcriptional factors for this process (Maguire et al., 2019). Notably, ABCA1, ABCG1, and SR-B1 control the removal of cholesterol and phospholipids out of macrophages by directing lipid droplets to apolipoprotein A1 and high-density lipoprotein (HDL) (Moore and Tabas, 2011; Maguire et al., 2019). Dysregulation of cholesterol homeostasis in macrophages results in lipid uptake disturbances, foam cell formation, and the progression of AS. LncRNAs in macrophages have been demonstrated to manage cholesterol loading and foam cell formation. Figure 2 shows a summary of these findings.

Generally, lncRNAs serve as miRNA sponges. Several studies have identified the intricate interplay between lncRNAs and microRNAs in macrophages that significantly affect the cell cholesterol metabolism and lead to foam cell formation and AS development. For example, nuclear-enriched abundant transcript 1 (NEAT1) (Chen et al., 2018; Wang L. et al., 2019) and growth arrest–specific transcript 5 (GAS5) (Ye et al., 2018) are involved in oxidative stress, lipid uptake, and inflammation by targeting miRNAs and may cause exacerbation of atherogenesis. However, RP5-833A20.1 (Hu et al., 2015), the cholesterol homeostasis regulator of miRNA expression (CHROME) (Hennessy et al., 2019), and the H19-imprinted maternally expressed transcript (H19) (Han et al., 2018) can reverse cholesterol metabolism disturbances and alleviate the inflammatory response.

GAS5 is capable of mediating macrophage polarization (Chi et al., 2019; Sun et al., 2017), apoptosis (Chen et al., 2017), and inflammation (Ye et al., 2018). GAS5 is found abundantly in atherosclerotic plaques after ox-LDL treatment. In THP-1 cells, enrichment of GAS5 suppressed the miR-211 expression, aggravating the inflammatory response and stimulating the expression of MMP, whose production in foam cells exacerbates proteolytic rupture of extracellular matrix components in plaque lesions. These findings support the role of GAS5 as a contributor of plaque destabilization in AS (Ye et al., 2018).

The nuclear lncRNA NEAT1 is a pro-atherosclerotic agent shown to serve as a sponge for downstream miR-128 (Chen et al., 2018) and miR-342-3p (Wang et al., 2019) targets. NEAT1 triggered an inflammatory response and oxidative stress by suppressing miR-128 in RAW264.7 cells after ox-LDL stimulation. Down-regulating NEAT1 repressed not only cell proliferation, inflammation, and the oxidative stress process but also inhibited CD36 expression, foam cell formation, and apoptosis (Chen et al., 2018). In addition, the NEAT1-miR-342-3p pathway modulates inflammation and lipid uptake. In THP-1 cells, lipid uptake was inhibited by NEAT1 silencing plus miR-34-3p mimics treatment. Because of this, NEAT1 blockade could suppress the ox-LDL–induced apoptosis and inflammation via miR-342-3p curbing (Wang et al., 2019).

Another nuclear lncRNA RP5-833A20.1 is anti-atherosclerotic. The expression of RP5-833A20.1 under ox-LDL or ac-LDL treatment weakens the expression of nuclear factor IA (NFIA) in THP-1-derived foam cells. In an ApoE^−/−^ mice model, NFIA overexpression enhanced HDL cholesterol (HDL-C), decreased the production of LDL cholesterol (LDL-C) and very LDL cholesterol (VLDL-C), and reduced the secretion of inflammatory cytokines in plasma. Meanwhile, NFIA promoted reverse cholesterol transport across cell membranes by stimulating ABCA1 and ABCG1 expression. ABCA1 and ABCG1 can deliver cholesterol across cell membranes (Stefulj et al., 2009) and may enable AS regression. Overexpressing RP5-833A20.1 and hsa-miR-382-5p mimics *in vitro* effectively down-regulated the expression of ABCA1 and ABCG1 but elevated the expression of SRA1, CD36, and NF-κB. As a result of this, lncRNA RP5-833A20.1 can stimulate cholesterol efflux in human macrophages and relieve the inflammatory response via the RP5-833A20.1- has-miR-382-5p pathway (Hu et al., 2015).

Furthermore, CHROME levels in the plasma and atherosclerotic plaques of patients with coronary artery disease (CAD) are elevated. Cells expressing wild-type CHROME *in vitro* inhibit the expression of miRNAs, such as miR-27b, miR-33a, miR-33b, and miR-128; these miRNAs suppress cholesterol efflux and prohibit HDL biogenesis.

CHROME derepresses these collective target genes through its miRNA interactions, which further affects cholesterol transport. Cells lacking CHROME express lower levels of ABCA1, cannot efflux cholesterol, and present reduced formation of nascent HDL particles in response to activating sterol-activated liver X receptor (LXR), leading to reduced expression of genes involved in the response to cholesterol excess in human hepatocytes and macrophages (Hennessy et al., 2019).

H19 participates in many pathological processes, including tumorigenesis (Ghafouri-Fard et al., 2020), cerebral ischemia-reperfusion injury (Wang et al., 2017), and acute myocardial infarction (Yu and Dong, 2018). H19 is highly expressed, while miR-130b is down-regulated in blood samples of patients with AS. This suggests that in ox-LDL stimulated RAW264.7 cells, H19 participates in adipogenesis and the inflammatory response by inhibiting the activity of miR-130b. miR-130b is a target for H19; H19-induced miR-130b expression after ox-LDL treatment stimulates anti-inflammatory cytokine production and decreases pro-inflammatory cytokine levels. Knocking down H19 using shRNAs alleviated lipid metabolism disturbances and decreased the inflammatory response by mitigating lipid accumulation and promoting the lipid metabolism (Han et al., 2018). H19 is also a regulator of hepatic inflammation during cholestasis that secretes exosome cargos in cholangiocytes (Li et al., 2020).

The plaque-enriched lncRNA in atherosclerotic and inflammatory bowel macrophage regulation (PELATON) lncRNA is a potential regulator of macrophage phagocytosis. After being confirmed by *in situ* hybridization, PELATON was enriched in unstable human atherosclerotic plaques with a necrotic core and plaque shoulders and colocalized with the M1 marker CD68. Knocking down PELATON in monocyte-derived macrophages markedly reduced the cell phagocytotic performance by reducing the CD36 mRNA (Hung et al., 2019). lncRNA E330013P06 (E33) also caused foam cell formation by up-regulating CD36 expression. Under type 2 diabetic (T2D) conditions, HG and PA treatment of macrophages greatly up-regulates the expression of E33. Exogenous expression of E33 highly induces inflammation by increasing inflammatory expression of Nos2, Il6, and Ptgs2 genes, along with foam cell formation through up-regulation of CD36 expression, resulting in pro-atherogenic macrophages responses (Reddy et al., 2014).

The pro-atherosclerotic taurine up-regulated gene 1 (TUG1) can worsen AS via the miR-92a/FXR1 axis (Yang and Li, 2020). TUG1 overexpression increases plaque size and enhances macrophage recruitment to the plaque area by targeting apolipoprotein (apo) M in ApoE^−/−^mice. Generally, ApoM is a critical lipocalin for delivering lipid sphingosine-1-phosphate (S1P). ApoM delivers S1P to its S1P receptors on endothelial cells. The anti-atherosclerotic ApoM can regulate high-density lipoprotein metabolism, protecting against oxidation and mediating CE (Christoffersen et al., 2011; Nádró et al., 2018). TUG1 was found to down-regulate ApoM levels via miR-92a inhibition and FXR1 stimulation in mouse liver NCTC 1469 cells. In RAW264.7 cells, TUG1 overexpression significantly decreased ABCA1 and ABCG1 expressions, which consequently slowed down the CE rate (Yang and Li, 2020).

In recent studies, GAS5 (Meng et al., 2020) and cyclin-dependent kinase inhibitor 2B antisense noncoding RNA (CDKN2B-AS1, also known as ANRIL) (Ou et al., 2020) showed an important role on AS development through its EZH2-mediated ABCA1 transcription targeting. In Meng’s work, GAS5 stimulated lipid accumulation and prevented cholesterol efflux by regulating ABCA1 in macrophage-derived foam cells (Meng et al., 2020).

By recruiting zeste homolog 2 (EZH2), one of the enzymatic factors of the polycomb repressive complex (Lu et al., 2018), to the promotor region of ABCA1, GAS5 transcriptionally represses its target genes (Shi et al., 2018). Knocking down GAS5 can greatly reverse cholesterol transportation and decrease lipid accumulation by alleviating the EZH2-dependent transcriptional inhibition of ABCA1. EZH2 enhances the triple methylation of lysine 27 (H3K27) in the promoter region of ABCA1. GAS5 transcriptionally inhibits ABCA1 by binding to the EZH2 enhancer. EZH2 can promote AS progression by efficiently blocking ABCA1 transcription in AS (Lv et al., 2016).

Similarly, the lncRNA CDKN2B-AS1 promotes cholesterol uptake and accumulation and inhibits macrophage reverse cholesterol transport (mRCT) in macrophage-derived foam cells (Ou et al., 2020). The detrimental effects of CDKN2B knockdown on atherosclerotic lesions could be reversed by sh-CDKN2B-AS1 in an *in vivo* mouse model. Mechanically, CDKN2B-AS1 can package an RNA–DNA triplex in the CDKN2B promoter region. This triplex can recruit EZH2 and CTCF to the promoter region of CDKN2B to increase histone methylation, which then adversely affects CDKN2B transcription (Ou et al., 2020).

In contrast, LincRNA-DYNLRB2-2, macrophage-expressed LXR-induced sequence (MeXis), HOXC cluster antisense RNA 1 (HOXC-AS1), and ANRIL are anti-atherogenic molecules that modulate cholesterol efflux and cholesterol accumulation. Among them, LincRNA-DYNLRB2-2 (Hu et al., 2014) and MeXis (Hennessy et al., 2019) both target ABCA1 to regulate the cholesterol metabolism. The ox-LDL–induced increased lincRNA-DYNLRB2-2 expression in THP-1 macrophage–derived foam cells ameliorates inflammation by up-regulating G protein–coupled receptor 119 (GPR119), meanwhile improving ABCA1-regulated cholesterol efflux via a glucagon-like peptide 1 receptor (GLP-1R) signaling pathway. GPR119 is an anti-inflammatory regulator that induces ABCA1 expression and can be controlled by GLP-1R–mediated signaling cascades. By inducing the expression of ABCA1, GPR119 promotes cellular cholesterol efflux through apoA-I. Therefore, lincRNA-DYNLRB2-2 prevents atherosclerotic plaque formation by repressing expression of inflammation and adhesion molecules and increasing cholesterol efflux (Hu et al., 2014). MeXis is an amplifier of the critical cholesterol efflux gene Abca1, whose transcription is mediated by LXR. In response to LXR signaling, MeXis expression induction in macrophages promotes the expression of Abca1 and consequently improves cholesterol efflux. MeXis knockout in the bone marrow of mice represses Abca1 and inflammatory gene expression, causing an impaired cell response to cholesterol overload and plaque development. The LXR-MeXis-Abca1 axis may reverse cholesterol delivery and play a protective role on AS development (Sallam et al., 2018; Xie Y. et al., 2019). Additionally, ANRIL could serve as a biomarker candidate of AS that is up-regulated in atherosclerotic plaques and in patients’ plasma (Hao et al., 2016). ANRIL also reduces inflammation and promotes cholesterol efflux by blocking ADAM10 expression. ADAM10 can shed or cleave several molecules on cell surface–like adhesion molecules and cytokines (van der Vorst et al., 2018). As a result of this, ANRIL suppresses the cytokine production and inflammation induced by AS, and it promotes cholesterol efflux (Li et al., 2019).

Moreover, the oncogenic LncRNA zinc finger NFX1-type containing one antisense RNA 1 (ZFAS1) is a viable target to ameliorate the development of AS because it reduces the cholesterol efflux rate and facilitates inflammation (Tang et al., 2020). In THP-1 macrophage-induced macrophages, overexpressing ZFAS1 promotes inflammatory responses and blocks cholesterol efflux. ZFAS1 is an upstream factor for miR-654-3p, which can target and suppress the expression of ADAM10 and RAB22A. In short, ZFAS1 can positively mediate the expression of ADAM10 and RAB22A by sponging miR-654-3p (Tang et al., 2020).

Lipid uptake, cholesterol esterification, and efflux are the main processes for the macrophage lipid metabolism. Disturbance of these processes leads to lipid accumulation and finally causes foam cell formation. lncRNAs participate in macrophage lipid metabolism and atherosclerotic plaque formation by targeting microRNAs or controlling the gene expression of important lipid metabolic enzymes or proteins.

HOXC cluster antisense RNA 1 (HOXC-AS1) exerts an inhibitory effect on ox-LDL–mediated cholesterol accumulation by improving homeobox C6 (HOXC6) expression in human THP-1 cells. HOX genes have significant regulatory effects on the cardiovascular system through vasculature function regulation (Miano et al., 1996). HOXC-AS1 and HOXC6 are both down-regulated in human atherosclerotic plaques. Lentivirus-mediated overexpression of HOXC-AS1 promoted the expression of HOXC6 and blocked ox-LDL–induced foam cell formation and disruption of cholesterol homeostasis in THP-1 cells. Ox-LDL could repress HOXC6 expression by reducing HOXC-AS1, partly suppressing Ox-LDL–mediated cholesterol accumulation to prevent AS (Huang C. et al., 2016).

Dysregulated lipid metabolism in macrophages is a potential cause of the foam cell formation, which speeds up the progress of AS. Several lncRNAs mentioned above play important roles during this process.

## Long Noncoding RNAs as Potential Biomarkers and Therapeutic Perspectives

Among the noncoding RNAs, many miRNAs have been identified as disease biomarkers for a variety of cardiovascular diseases (CVDs) (Indolfi et al., 2019). Advances in the field of lncRNAs have provided evidence that these molecules display specific characteristics that make them attractive as prospective therapeutic targets to be exploited for clinical use (Indolfi et al., 2019). The expression profiles of lncRNAs have revealed individual lncRNAs or clusters of lncRNAs within plaque lesions. These molecules are potential biomarker candidates and can be seen as prospective therapeutic targets during AS progression (Fasolo et al., 2019; Indolfi et al., 2019).

On the one hand, as potential biomarkers, some “sponge” lncRNAs are more robust than miRNAs. LncRNAs can be upstream targets of miRNAs. For example, the macrophage anti-atherosclerotic lncRNA ZFAS1 serves as sponge that binds to miR-654-3p and improves the expression of ADAM10 and RAB22A (Tang et al., 2020). Moreover, some lncRNAs “sponges” are capable of interacting with more than one miRNA. CHROME can exert regulatory roles on cholesterol efflux and reverses cholesterol transport by reducing the expression of four miRNAs, namely, miR-27b, miR-33a, miR-33b, and miR-128 (Hennessy et al., 2019). Several miRNAs are regulated by a single lncRNA; therefore, targeting such lncRNAs can be convenient (Indolfi et al., 2019).

On the other hand, lncRNAs can be biomarkers to predict different AS stages. RAPIA, for instance, is highly expressed in advanced atherosclerotic sites in mice models. The high level of RAPIA expression in atherosclerotic plaques may predict the occurrence of advanced AS (Sun et al., 2020). Suppression of RAPIA showed similar effects to those of atorvastatin on advanced atherosclerotic plaques by attenuating lipid accumulation and decreasing plaque size in advanced atherosclerotic plaques of mice models. RAPIA has been seen as a promising therapeutic target for treating advanced atherosclerotic lesions, especially in patients resistant or intolerant to statins.

LncRNAs offer the promise of a noninvasive diagnostic tool to manage AS. These molecules can be detected in the patients’ sera and are considered attractive disease biomarker candidates (Zeng et al., 2018). ANRIL is a potential AS biomarker as it was found to increase in human atherosclerotic plaques and plasma (Hao et al., 2016). In a clinical study, GAS5 was specifically down-regulated in the sera of patients with CAD (Yin et al., 2017): GAS5 expression was greatly down-regulated in patients with CAD compared to its levels in normal controls and in patients with diabetes mellitus (the expression levels of GAS5 were decreased only in patients with CAD) (Indolfi et al., 2019). The expression of CHROME was also found to be high in the sera of patients with CAD and atherosclerotic plaques (Hennessy et al., 2019). Thus, significant high levels of these candidate lncRNAs in the blood may be predictors of CAD.

## Future Perspectives and Conclusion

As Koelwyn stated, identifying the changing patterns of metabolism in monocytes and macrophages during early atherogenesis and figuring out at which point these processes become maladaptive and progress to the next stage are important. The progression of advanced AS can trigger acute cardiovascular diseases, like heart attack and stroke. Understanding the changing metabolism of macrophages across all stages of AS development is urgent, as is identifying the timing of early foam cell lesion formations, the progression to advanced plaques, and the rupture of vulnerable plaques (Koelwyn et al., 2018). Several lines of evidence have suggested that lncRNAs could be involved in regulating macrophage inflammation, macrophage cholesterol loading, macrophage differentiation, polarization, and apoptosis during the development of AS. Additionally, lncRNAs are promising candidates for AS biomarkers and potential therapeutic targets, like ANRIL, which was found to be greatly increased in patients’ plasma (Hao et al., 2016), or the fact that blocking RAPPIA can partially mimic the effect of atorvastatin (Sun et al., 2020). We believe macrophage-related lncRNAs provide a novel and unique perspective to investigate the crossroads between chronic inflammation and AS. Future studies need to provide insights into the lncRNAs’ functions in macrophages and explain how they function in the development of AS.
