# Corrigendum: Functioning of Long Noncoding RNAs Expressed in Macrophage in the Development of Atherosclerosis

**DOI:** 10.3389/fphar.2021.767272

**Published:** 2021-10-11

**Authors:** Xirui Ma, Huifang Liu, Fengling Chen

**Affiliations:** Department of Endocrinology and Metabolism, Shanghai Ninth People’s Hospital, Shanghai Jiao Tong University School of Medicine, Shanghai, China

**Keywords:** long noncoding RNA, Atherosclerosis, macrophage, NF-kappa B, foam cell macrophage

In the original article, we neglected to include the funder “The National Natural Science Foundation of China, No. 81670735 to FC, The National Natural Science Foundation of China, No. 81400802 and Outstanding Youth Training Project from Shanghai Ninth People’s Hospital the National Natural Science Foundation of China, jyyq 08201607 to HL”.

The authors apologize for this error and state that this does not change the scientific conclusions of the article in any way. The original article has been updated.

